# Cardiac PPARα Protein Expression is Constant as Alternate Nuclear Receptors and PGC-1 Coordinately Increase During the Postnatal Metabolic Transition

**DOI:** 10.1155/2008/279531

**Published:** 2008-01-17

**Authors:** Norman E. Buroker, Xue-Han Ning, Michael Portman

**Affiliations:** Department of Cardiology, Children's Hospital and Regional Medical Center, 4800 Sand Point Way N.E., Seattle, WA 98105, USA

## Abstract

Gene expression data obtained in mouse heart indicate that increased expression for the nuclear receptor, peroxisomal proliferator activated receptor α (PPARα), prompts the postnatal transition from predominantly carbohydrate to fatty acid oxidation preference. However, no phenotypic or proteomic data are available to confirm downstream signaling and metabolic transition in mice. We studied the hypothesis that shifts in nuclear receptor expression trigger the newborn metabolic switch in a newborn sheep. This species is well characterized with regards to developmental changes in substrate oxidative metabolism. Heart tissues from fetal (130 days gestation), newborn ≤24 hours, and 30-day old lambs were evaluated for protein expression from multiple enzymes controlling oxidative metabolism as well as principal nuclear receptors and coactivators. Although muscle and liver type carnitine palmitoyl transferases I showed no significant changes to correspond to the metabolic transition, hexokinase II protein content showed a profound transient drop, and pyruvate dehydrogenase kinase steadily increased. PPARα showed no increases preceding or during the transition, while peroxisomal proliferator activated receptor gamma coactivator-1 (PGC-1) increased approximately 20-fold transiently in newborn heart in conjunction with significant increases in thyroid hormone receptor α1 and retinoid-activated receptor α. These data challenge the paradigm that increases in PPARα prompt the postnatal metabolic switch, and suggest that other nuclear receptors play a major role. As thyroid hormone (TH) modulates PGC-1 expression in sheep during development, these data further suggest that well-characterized perinatal TH surge in sheep contributes to this metabolic switch.

## 1. INTRODUCTION

The fetal heart uses glucose and lactate as the main oxidative substrate sources, then switches to fatty acids as the predominant fuel shortly after birth [1]. Rapid expansion of mitochondrial pool as well as total oxidative capacity accompanies the perinatal metabolic transition. Initial newborn suckling likely stimulates release of hormonal factors, which trigger modification of substrate preference. Recent data indicate that multiple nuclear receptors and their coactivators initiate transcriptional events controlling both newborn substrate switching and perinatal mitochondrial biogenesis.

The nuclear receptor family includes classical endocrine receptors activated by ligands such a thyroid hormone or steroid hormones. Other more recently identified nuclear receptors (NR) respond to dietary-derived lipid intermediates involved in the metabolism of these activating ligands. In particular, the fatty acid-activated peroxisome proliferator-activated receptors (PPAR) participate as key regulators of cardiac energy metabolism. The hormones or ligands for the nuclear receptors attach to their respective ligand binding domains. These receptors will attach to DNA response elements (REs) of their target genes as monomers, homodimers, or partner as heterodimers [2, 3]. Many NRs involved in metabolic regulation heterodimerize with retinoid X receptor, creating a potential mechanism for regulatory integration. Ligand binding promotes a permissive receptor conformation for coactivator interaction. Coactivators such as PPAR*γ* coactivator-1 (PGC-1) bind the receptor in the process of establishing a transcriptional complex with RNA polymerase-2 to initiate transcription of target genes [3–5]. PGC-1 enhances transactivation mediated by numerous nuclear receptors involved in energy metabolism and mitochondrial biogenesis, thereby providing a second mechanism for integration of these processes.

Although NR-mediated control of cardiac energy metabolism has been examined with gain or loss of function using transgenic models, developmental regulation of these processes has not been studied in detail. Messenger RNA expression studies have provided the prevailing evidence for the PPAR and PGC-1 triggering of the cardiac metabolic switch after birth. However, multiple NRs are subject to several post-transcriptional processes including alternate heteronuclear RNA splicing and differing translation site initiation, as well as end-product feedback inhibition. For the most part, regulatory patterns have not been confirmed at the protein expression level. Accordingly, we studied developmental integration and coordination of protein expression for three major nuclear receptor families involved in regulation of both cardiac metabolism and mitochondrial biogenesis: thyroid hormone receptors (TRs), PPARs, and RXRs. This study included analyses of PGC-1, as it provides a coactivator function for both TRs and PPARs. Furthermore, previous studies involving NR regulation of developmental metabolic switching have been performed in mice, a poorly characterized species with regard to newborn cardiac metabolism [6]. Since substantial data is available defining developmental cardiac energy metabolism in sheep, we used this species to test the hypothesis that nuclear receptor signaling prompts the newborn metabolic switch. The data obtained in this well-characterized species challenge existing paradigms regarding triggering for the postnatal metabolic transition.

## 2. MATERIALS AND METHODS

### 2.1. Animal samples

Domestic sheep (Ovis aries) were used for our study. Heart samples were collected at 130 days into gestation (F), one day after birth (N), and 30 days after birth (C) (n=6, from each group). Heart tissue from the left ventricle was quickly blotted dry, frozen in liquid nitrogen, and stored at −80°C. Our investigation conforms to the *Guide for the Care and Use of Laboratory Animals* published by the US National Institutes of Health (NIH Publication No. 85-23, revised 1996). The Animal Care Committee of the University of Washington approved all animal protocols.

### 2.2. Protein isolation

The frozen heart tissue was diced and homogenized at 4°C in a three-fold amount of protein lyses buffer (50 mM Tris-Cl, pH 8.0, 100 mM NaCl, 20 mM HEPES, pH7.9, 1% Triton X-100, 10 mM NaF, 1 mM Na_3_ VO_4_, 100 ug/ml C_7_ H_7_ FO_2_S, 5 ug/ml Aprotinin, and 5 ug/ml Leupeptin). The samples were then incubated on ice for 30 minutes and centrifuged at 10,000 rpm for 10 minutes at 4°C. The supernatant was transferred to another microfuge tube and centrifuged one more time. The supernatant was stored at −80°C for immunoblotting analysis.

### 2.3. Immunoblotting

Fifty micrograms of total protein extracts from sheep heart tissue were electrophoresed along with one lane containing thirty micrograms of human HeLa cells as a positive control and one lane of molecular weight size markers (Chemichrome Western Control, Sigma-Aldrich Co., Mo, USA) in a 4.5% stacking and 7.5, 10, or 12% running SDS-polyacrylamide gel depending on the molecular weight of the protein of interest. The gels were then electroblotted onto polyvinylidene fluoride (PVDF) plus membranes. The western blots were blocked for one hour at room temperature with either a 1% or 5% nonfat milk (depending on the antibodies requirements) in Tris-buffered saline plus Tween-20 (TBST)[10 mM Tris-HCl, pH 7.5, 150 mM NaCl, and 0.05% Tween-20], followed by overnight incubation at 4°C with each primary antibody diluted in the appropriate blocking solution as recommended by the supplier. The primary antibodies used in the study are *β*-actin (SC-1616), HK2 (SC-6521), MTCO3 (SC-23986), PGC-1 (SC-5814), PPAR*α* (SC-9000), and RXR*α* (SC-553) obtained from (Santa Cruz Biotechnology, Inc., Calif, USA). The primary antibody TR*α* (PA1-211A) was obtained from (Affinity BioReagents, Inc., CO, USA). The primary antibodies L&M-CPTI and PDK2 were obtained as personal gifts from Gebre Woldegiorgis and Robert Harris, respectively. After two five-minute washes with TBST and one five-minute wash with Tris-buffered saline (TBS), membranes were incubated at room temperature for one hour with the appropriated secondary antibody conjugated to horseradish peroxidase (HRP). The membranes were washed twice for ten minutes with TBST and visualized with enhanced chemiluminescence after exposure to Kodak biomax light ML-2 film. The membranes were stripped by washing them two times for 30 minutes with 200 mM Glycine, 0.1% SDS, and 1% Tween-20 (pH adjusted to 2.2), followed by three ten-minute washes with TBS. The membranes were again blocked for one hour as above, followed by overnight incubation at 4°C with *β*-Actin antibody diluted 1 : 200 in blocking solution. The next day, the membranes were washed (as above), the appropriate secondary-HRP antibody was applied, and the remaining procedures (as described above) were followed. The *β*-actin was used to verify protein lane loadings.

### 2.4. Statistical analysis

**Figure 1:**
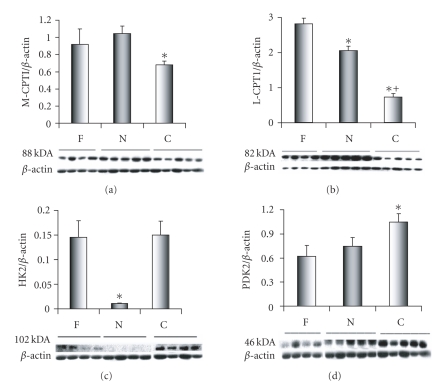
Immunoblots and expression patterns for enzymes controlling substrate oxidation. Data is shown for left ventricle from three development periods (F, N, C) as defined in text. Abbreviations are carnitine palmitoyltransferase I (muscle isoform M-CPTI), carnitine palmitoyltransferase I (liver isoform L-CPTI), hexokinase 2 (HK2), and pyruvate dehydrogenase kinase 2 (PDK2). M-CPTI protein expression decreases occurred between N and C (*P<.01). L-CPTI protein expression differences decreased immediately after birth F (*P<.01) and continued to drop in C (^+^ P<0.001, versus N). HK2 protein expression exhibited a marked but transient decrease after birth N (*P<.05, versus F & C). PDK2 protein expression increased with significance noted in C compared to F and N (*P<.05, F & N).

The film expression was determined using the ImageJ 1.32j program produced by Wayne Rasband for (the National Institute of Health, Md, USA). The protein expression was standardized against *β*-Actin and the means and standard errors for the three stages of heart development are displayed in a histogram (Figure 1). Statistical significance was determined with Student's t-test (two tailed) comparison between all stages of development.

## 3. RESULTS

### 3.1. Carnitine palmitoyltransferases

The enzyme carnitine palmitoyltransferase I (CPT I; palmitoyl-CoA:L-carnitine O-palmitoyltransferase; EC 2.31.21) is a rate-limiting step in mitochondria transport during FA oxidation. It catalyzes the initial reaction of acyl-CoA and carnitine to acylcarnitine during the mitochondria import of long-chain FAs into the inner mitochondria membrane. PPAR regulates CPTI and turns the gene on during the FA oxidation cascade of events shortly after birth [7, 8]. In mammalian heart, there are two CPTI isoenzymes: a liver, L-CPTI isoenzyme, also known as CPTI*α*: and a muscle, M-CPTI isoenzyme, also known as CPTI*β* [9, 10] In human tissue, the L-CPTI protein is composed of 773 amino acids that corresponds to a molecular weight of 88.3 kDa [P50416]. In sheep heart ,the L-CPTI isoenzyme corresponds to a molecular weight of 82 kDa (Figure 3), which is smaller than that reported in human tissue, but in agreement with what has been reported in fetal and newborn lambs [9]. In human tissue, the M-CPT I protein is composed of 772 amino acids which corresponds to a molecular weight of 87.8 kDa [Q92523]. The adult sheep protein sequence is highly conserved with relation to other mammals with an 89% similarity to humans and 88% similarity to mouse and rat [11]. In sheep heart, the M-CPTI antibody recognizes an 88-kDa isoenzyme consistently expressed in the three study groups (Figure 1). The 88-kDa protein corresponds to the 771 amino acids reported for M-CPTI in sheep [C81315], but is in disagreement with the 80-kDa protein that has been previously reported in sheep [9]. In this study, the M-CPTI (Figure 3(a)) and L-CPTI (Figure 3(b)) protein expression was significantly greater in the day old samples then in the 30-day old samples, while the L-CPTI protein expression was significantly greater in the fetal then in either the day old or 30-day old samples (Figure 1).

### 3.2. Hexokinase

Hexokinase (HK, E.C.2.7.1.1) in mammalian tissues exist as four isoenzymes (HK1-4) with distinct kinetic properties and tissue distribution [12]. The hexokinases are rate limiting glycolytic enzymes that catalyze the phosphorylation of glucose to glucose-6-phosphate [13]. HK type 2 and HK type 4 are found in heart tissue [14] . In sheep heart, the HK2 antibody detects a 102-kDa protein among the three study groups (Figure 1). The 102-kDa protein corresponds to the molecular weight of the human HK2 isoenzyme with 917 amino acids [P52789]. A 102-kDa protein was also detected by this antibody in a HeLa human cell line obtained from (Santa Cruz Biotechnology, Inc., Calif, USA) (data not included). In this study, we monitored the appearance of HK2 during the three stages of sheep development and found a significant decrease in HK2 protein expression for the day old samples compared to either the fetal or 30-day old samples. This finding indicates that a reduction in glycolysis at birth due to the onset of FAO.

### 3.3. Pyruvate dehydrogenase kinase

The isoenzyme pyruvate dehydrogenase kinase 2 (PDK2; pyruvate dehydrogenase [lipoamide] kinase isoenzyme 4; EC 2.7.1.99) is one of four PDK isoenzymes found in mammalian tissues. PDK2 is expressed at high levels in heart tissue [15–17] and is regulated by PPAR*α* [18]. The enzyme is responsible for phosphorylation of pyruvate dehydrogenase (PDH), a mitochondrial multienzyme complex, rendering it inactive. The PDH catalyzes the oxidative decarboxylation of pyruvate, linking glycolysis to the tricarboxylic acid cycle and FA synthesis [18]. Increased levels of PDK in early postnatal life for PDH inactivation are thought to be the result of changes in lipid supply and a switch from glucose to FAs as an energy supply [19]. In addition to hexokinase, PDK is also a rate-limiting step in the glycolytic pathway [20, 21]. In human tissue, the PDK2 isoenzyme is composed of 407 amino acids which corresponds to a molecular weight of 46 kDa for the isoenzyme [Q15119]. PDK2 has its strongest expression in the day old (N) sheep samples. In sheep heart, the PDK2 antibody detects a 46-kDa protein among the three study groups (Figure 1). A 47-kDa protein was also detected by this antibody in a HeLa human cell line obtained from (Santa Cruz Biotechnology, Inc., Calif, USA) (data not included). In this study, PDK2 protein expression levels were significantly greater in the 30-day old (C) samples when compared to either fetal (F) or the one-day old (N) samples; (Figure 1) indicating the switch from glucose to FAO.

### 3.4. Mitochondrial proteins involved with cardiac energy metabolism

**Figure 2:**
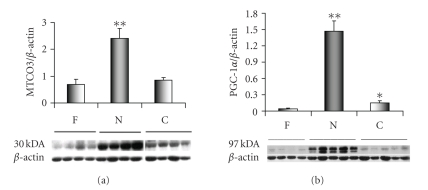
Cytochrome c oxidase 3 expression serves a reporter for the mitochondrial genome (MTCO3; Cytochrome c oxidase polypeptide 3; E.C. 1.9.3.1). MTCO3 increased transiently in the newborn (**P<.01, versus F & C). Coordinate changes in protein content occurred for PGC-1*α*, implicated as a regulator of mitochondrial biogenesis.

With the beginning of aerobic development at birth there is a rapid deployment of new mitochondria in cardiac cells to handle the FA energy metabolism and ATP output [22]. The mitochondrial genome must be running near full capacity with genome replication for new mitochondria as well as the transcription of mitochondria genes [23] in order to accommodate this expansion. We used protein expression for the cytochrome c oxidase 3 as a reporter for mitochondrial biogenesis. Cytochrome c oxidase 3 (MTCO3; Cytochrome c oxidase polypeptide 3; E.C. 1.9.3.1) is one of three subunits transcribed in the mitochondria from a total of 13 subunits that make up cytochrome c oxidase. The remaining ten subunits are transcribed from nuclear genes [23]. In sheep heart, the MTCO3 antibody detects a 30-kDa molecular weight protein, which represents the cytochrome c oxidase 3 in the three study groups. MTCO3 protein expression is greater in N then either the F or C (Figure 2). We did not see comparable changes for the nuclear cytochrome c oxidase, subunit 4, (NCO4) gene (data not included). This difference between the expression of mitochondria and nuclear cytochrome c oxidase subunits during mitochondria biogenesis has previously been reported [24].

### 3.5. Peroxisome proliferator-activated receptor-*γ* co-activator 1 (PGC-1)

In sheep, the PGC-1 antibody recognizes the PGC-1*α* protein or the 91-kDa isoform, while the PRC and PGC-1*β* isoforms were not detected among the three study groups (Figure 2). PGC-1*α* displays a significant increase in protein expression in the day old samples (N) compared with either the fetal (F) or 30-day old samples (C), while the 30-day old samples (C) were found to have a significantly greater protein expression level than the fetal samples (F). In a previous sheep study, we noted that PGC-1 protein expression was near threefold greater in C than in F.

### 3.6. Peroxisome proliferator-activated receptors (PPAR*α*)

**Figure 3:**
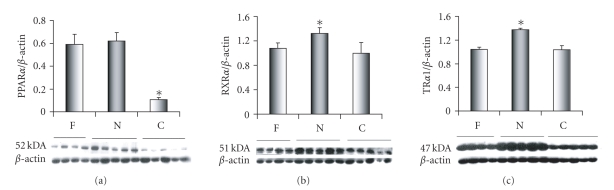
Content for nuclear receptors peroxisome proliferator-activated receptor (PPAR*α*), (b) retinoid X receptor alpha (RXR*α*), and thyroid hormone receptor (TR*α*_1_) among three stages (F, N, C). Modest transient but significant elevations for RXR*α* and TR*α*_1_ occurred in N compared to both F and C (*P<.01). PPAR*α* expression did not change immediately after birth, but later decreased compared to F and N (*P<.01).

The PPAR*α* antibody detects the 52-kDa nuclear receptor at each stage of development (Figure 3). In human tissues, the PPAR*α* protein is composed of 478 amino acids that reflects a molecular weight of 52 kDa [Q07869], which coincides with the molecular weight observed in sheep. The 52-kDa protein was also detected by this antibody in a HeLa human cell line obtained from (Santa Cruz Biotechnology, Inc., Calif, USA) (data not included). The protein expression of the PPAR**α** nuclear receptor was found to be significantly lower in the 30-day old samples (C) when compared to the Fetal (F) and the day old samples (N) (Figure 3).

### 3.7. Retinoid X receptors (RXR*α*)

In sheep heart, the RXR*α* antibody detects the 51-kDa nuclear receptor at each stage of development (Figure 3) corresponding to the full length RXR*α* is 462 amino acids, which represents a molecular weight of 51 kDa [P19793]. We noted a small but significant surge in RXR*α* expression in the one-day old sheep heart (Figure 2(b)).

### 3.8. Thyroid hormone receptor (TR*α*_1_)

In sheep heart, the TR*α*1 antibody detects the 47-kDa nuclear receptor among the three study groups (Figure 3). In humans and rodents, the full length of TR*α* is 410 amino acids, which corresponds to a molecular weight of 47 kDa. In this study, the THR*α*1 nuclear receptor has a significantly greater level of protein expression in the day old samples (N) compared with either the fetal (F) or the 30-day old samples (C) (Figure 3).

### 3.9. Other nuclear receptor isoforms and metabolic proteins of interest

Antibodies against other protein expression were used. These include PPAR*β*, PPAR*γ*, RXR*β*, TR*α*2, TR*β*, MYLCD, and PDK4. Unfortunately, a cross reactivity exists between the secondary antibodies (i.e., those with goat, mouse, and rabbit hosts for the primary antibodies) and the one-day and 30-day old stages of development in our sheep samples. Consequently, we could not get reliable data for these proteins of interest.

## 4. DISCUSSION

Several investigations have established the time course for maturation of cardiac energy metabolism in the sheep model in vivo. For instance, Bartelds et al. [1] showed that glucose and lactate were the prime energy substrates during ovine fetal life, and the switch to fatty acids as prime oxidative substrate occurred within 2–16 days after birth [1, 25]. Our laboratory has shown that regulation of myocardial oxidative phosphorylation matures within the same age period in parallel with accumulation of the adenine nucleotide translocator protein [26]. Considered in summation, these studies indicate that postnatal transitions in oxidative phosphorylation and substrate oxidation occur coordinately and their regulation is integrated through a unifying signaling mechanism. The nuclear receptors, operating in conjunction with their coactivators, offer a potential mechanism for rapidly integrating these processes shortly after birth.

Fatty acid and carbohydrate metabolism generally exhibit reciprocal type regulatory patterns, where one decreases as the other increases [27]. Prior work in the newborn sheep heart has focused on carnitine palmitoyl transferase I as the pivotal enzyme determining the preferential shift for fatty acid oxidation over carbohydrate utilization. Bartelds et al. [1] detected postnatal increases in CPTI activity and protein content, which were much lower than the increase in the rate of LC-FA oxidation in vivo in the same animals. Furthermore, they found relatively high rates of CPTI activity in fetal lambs [9]. These data led to their contention that substrate supply was the major determinant for the increase preference for LC-FA oxidation around birth. Similarly, our data show steady decline in L-CPTI immediately after birth with maintained M-CPTI protein levels, and imply that CPTI activity or content does not regulate the postnatal metabolic transition in sheep. Studies in rabbit heart have also shown that the postnatal increases in fatty acid flux do not relate to CPTI-content, isoform pattern or activity. The increase in fatty acid oxidation in the rabbit heart relates directly to a reduction in levels of malonyl-CoA decarboxylase (MYLCD), an inhibitor of CPTI. PDH activity also increases in postnatal rabbit myocardium despite decreases in glucose oxidation.

In our sheep studies, we show for the first time changes in ovine expression for myocardial hexokinase 2 during development. This enzyme catalyzes glucose phosphorylation, a rate limiting step in glucose oxidation and positioned upstream from pyruvate dehydrogenase. Although, glucose transport across the sarcolemma is controlled in part by glucose transporters (GLUT1 and GLUT4), which are expressed abundantly in the fetal sheep heart [28], hexokinase-2 has emerged recently as the rate limiting glucose oxidation step during periods of stress [21, 29]. We also found modest but late postnatal increases for PDK2, which are consistent with inhibition of pyruvate dehydrogenase flux. Thus, our data imply that inhibition of the glucose oxidation pathway contributes to the postnatal metabolic switch with reciprocal and indirect activation of fatty acid oxidation accompanying.

The paradigm of PPAR*α* activation serving as the primary signaling mechanism for the postnatal myocardial metabolic switch has been propagated solely by studies demonstrating increases in PPAR*α* and PGC-1 mRNA levels in mice or rats during development [4, 30]. Supportive developmental expression studies for the corresponding proteins or their activity are lacking in the literature for these species. Furthermore, no published data, that we are aware of, is available regarding the occurrence and timing of the postnatal metabolic switching in either of these species. Extensive literature search reveals no studies regarding myocardial substrate oxidation patterns near or after birth in these species. Rather, mRNA data from rodents has been extrapolated to describe signaling for postnatal phenomenon characterized in larger species such as sheep, rabbit [31], and pigs [32–34]. This interpretative approach is of dubious value, as we have previously shown that mRNA levels for these nuclear receptors and coactivators demonstrate no coordination with protein content in the postnatal period [35]. Results from previous work in sheep imply that steady-state mRNA levels from PPAR*α* and PGC-1 are subject to auto feedback from respective proteins. The dramatic changes in content for the proteins controlling cardiac substrate and oxidative metabolism, such as hexokinase-2, and cytochrome c oxidase, are not preceded by elevation in PPAR*α* protein abundance. Hence, our study indicates that PPAR*α* protein content plays no role in initiating the immediate postnatal upregulation of these metabolic proteins.

Though the data challenge the concept that postnatal increases in PPAR*α* steady-state mRNA coordinates the metabolic shift, they do not eliminate a role for enhanced PPAR*α*-mediated transactivation of target genes. In the current model, PPAR*α* activity might be increased through simultaneous coactivation by PGC-1*α*, heterodimer formation through increased RXR availability, and enhanced ligand availability generated by newborn suckling and fatty acid intake. However, we have little downstream evidence for increased PPAR*α* transcriptional activity. On the contrary, known PPAR*α* targets, such as LCPTI, MCPTI, and PDK2 are not overtly elevated in terms of protein content during the immediate period after birth. Finally, hexokinase-2, rapidly upregulated by the PPAR*α* antagonist WY14643 in mice [36], shows a marked depression in this model.

PGC-1 remains an attractive candidate as a primary regulator for the postnatal myocardial transition, as this factor also appears to coordinate cross-talk between mitochondrial and nuclear genomes during development [24]. We have previously shown that gene and protein expression for nuclear-encoded adenine nucleotide translocator increases in sheep heart by 28–30 days after birth [26]. In the current study, we demonstrate that the mitochondrial encoded respiratory chain component, MTCO3 increases near four-fold in conjunction with the robust change in PGC-1. As PGC-1 and MTCO3 change coordinately, the data imply that PGC-1 coordinates substrate switching along with mitochondrial membrane expansion by affecting the mitochondrial genome. PGC-1 also closely links to total functional cytochrome c oxidase (Cyt *aa*_3_) and cytochrome c (Cyt c) content and respiratory capacity during postnatal ovine heart development [35]. However, we found no concomitant change in the nuclear-encoded cytochrome c oxidase, subunit 4. This finding suggests that content for nuclear-encoded components are adequate in the fetus, but that rapid postnatal mitochondrial biogenesis depends on PGC-1 promotion of mitochondrial encoded components.

This study was designed to sample three points during sheep development, which would determine the temporal relationship between protein expression and the well-documented late fetal and immediate postnatal surge in circulating thyroid hormone [37]. Since, multiple changes in hormone concentrations and environment occur during this time period, we cannot prove without a doubt that this change in thyroid hormone homeostasis exclusively causes increases in PGC-1 and nuclear receptors. However, thyroidectomy immediately after birth abrogates the postnatal thyroid hormone surge, reduces PGC-1*α* levels [35], and attenuates expansion of mitochondrial membrane protein content and respiratory capacity [38]. This observation supports the contention that thyroid hormone plays an important role in signaling the postnatal metabolic transition in heart. Our data from prior work and the current study suggest that T_3_ simultaneously elevates PGC-1*α*, and binds as a ligand to the TRs, which also exhibit increased expression, during this critical developmental period. These coordinated events would lead to increased transactivation of metabolic target genes by TRs and PPARs when binding as heterodimers with RXRs.

In summary, our data challenge specific concepts regarding the importance of PPAR*α* protein expression in control of the postnatal metabolic switch. First, we showed that alterations in hexokinase-2, a rate limiting step in glucose oxidation, accompany the transition. These data suggest that inhibition of glucose oxidation reciprocally stimulates fatty acid flux, as opposed to a direct increase in enzyme expression and /or activity of CPTI. Secondly, we demonstrate that no increase in PPAR*α* protein occurs prior to the postnatal metabolic switch in sheep. Thus, transcriptional mediation of this protein does not trigger the change in substrate preference. However, a robust postnatal increase in PGC-1 and the PPAR*α* binding partner, RXR*α*, does provide conditions for enhanced PPAR*α* activity.
